# Associations between diet and physical activity and mental health among Catalan youth

**DOI:** 10.1186/s13690-026-01899-y

**Published:** 2026-03-26

**Authors:** L. Serra, G. Renart-Vicens, L. Vall-llosera Casanovas

**Affiliations:** 1 Research Group on Statistics, Econometrics and Health (GRECS), University of Girona, Girona, 17004 Spain; 2 Center for Biomedical Research in Epidemiology and Public Health (CIBERESP), Madrid, Spain

**Keywords:** Mental health, Anxiety and depression, Physical activity, Dietary habits, Adolescents and young adults, Public health, Population-based survey, Social support

## Abstract

**Background:**

Mental health problems are increasingly prevalent among adolescents and young adults and represent a major public health challenge closely aligned with the United Nations Sustainable Development Goal 3 (Good Health and Well-being). Lifestyle-related and social factors, such as diet, physical activity, and social support, are closely related to emotional well-being during this critical life stage. This study examines the association between dietary habits, physical activity, and mental health among Catalan adolescents aged 15 to 25 years in Catalonia, within a population-based public health framework.

**Methods:**

Data were drawn from the 2022–2023 Catalan Health Survey (ESCA), including 855 participants aged 15–25. Weighted logistic regression analyses were used to assess associations between clinically diagnosed anxiety and depression and key personal and behavioral characteristics including age, sex, physical activity, social support, and diet.

**Results:**

Overall, 85.7% anxiety-free and 93.2% depression-free. Poorer mental health status was associated with female sex, older age, and frequent consumption of red or processed meats. In contrast, regular vigorous physical activity and strong social support were consistently associated with higher likelihood of reporting good mental health.

**Conclusion:**

These findings underscore the relevance of modifiable lifestyle and social determinants in promoting mental well-being among young people. From a public health perspective, interventions that encourage physical activity, healthy dietary habits, and social connectedness may contribute to advancing Sustainable Development Goal 3 by reducing the burden of anxiety and depression in youth populations.

**Table:** 

Text box 1. Contributions to the literature
• This study provides recent population-based evidence on mental health among adolescents and young adults in Catalonia, an age group that remains underrepresented in European public health research.
• It integrates lifestyle behaviors and social determinants within a single analytical framework, highlighting how physical activity, diet, and social support jointly relate to anxiety and depression.
• The findings contribute to understanding gender differences in the associations between physical activity and mental health during youth.
• By using representative survey data, the study informs prevention-oriented public health strategies aligned with Sustainable Development Goal 3.

## Introduction

Mental health issues among adolescents and young adults represent a growing public health concern worldwide. According to the World Health Organization (WHO), adolescence is defined as the period between 10 and 19 years of age, while youth encompass individuals aged 15 to 24 years [20]. This developmental stage is characterized by profound physical, emotional, and social changes that increase vulnerability to psychological difficulties such as anxiety and depression.

During the transition from adolescence to emerging adulthood, young people are particularly vulnerable to psychological risk factors such as academic stress, social pressure, and biological changes [20]. In this context, healthy lifestyle habits—such as regular physical activity and balanced nutrition—have been identified as potential protective factors that are associated with greater resilience and emotional well-being. A growing body of evidence indicates that regular physical activity is associated with lower levels of symptoms of anxiety and depression and improves self-esteem and cognitive function among adolescents and young adults [3, 11, 17].

In parallel, the relationship between diet and mental health has gained increasing attention. Studies have found that high consumption of red and processed meats is associated with higher odds of depression [10]. Cross-sectional evidence also suggests that a high intake of ultra-processed foods is associated with higher odds of depressive or anxious symptoms [8]. Although the biological mechanisms underlying these associations remain under debate, inflammation, oxidative stress, and gut microbiota alterations have been proposed as mediators [6]. Conversely, Mediterranean-type dietary patterns rich in fruits, vegetables, fish, and olive oil have been associated with better mental health [9].

The present study, therefore, examines how physical activity, dietary habits, and social support relate to anxiety and depression among Catalan adolescents and youth aged 15 to 25 years—an age range that overlaps the WHO definition of youth and captures the transition between adolescence and early adulthood.

In Catalonia, few studies have integrated these behavioural and social factors within a comprehensive framework to analyse mental health among young people.

## Methods

### Sample

This cross-sectional study draws on data from the 2022–2023 waves of the Catalan Health Survey (Enquesta de Salut de Catalunya, ESCA^1^), a continuous, population-based survey of non-institutionalized residents in Catalonia. ESCA is conducted by the Catalan Department of Health with technical support from the Catalan Statistical Institute and uses a probabilistic, multistage, stratified sampling design. Functional health sectors are selected first, followed by municipalities with probability proportional to population size, and finally individuals randomly selected from the municipal population register and stratified by age group and sex. Data are collected throughout the year via standardized computer-assisted personal interviews (CAPI).

ESCA is designed to be representative of the Catalan non-institutionalized population by sex, age groups, social class, and educational attainment. Because smaller municipalities are oversampled, survey weights are provided to restore representativeness. All analyses in the present study applied these sampling weights to ensure representativeness of individuals aged 15–25 years.

The full ESCA survey includes individuals across the entire age spectrum, from infancy to old age. For the purposes of this study, only respondents aged 15–25 years were retained. A complete-case approach was used: participants with missing data on the mental health variables (clinically diagnosed anxiety or depression) or on key personal and behavioural characteristics (physical activity, dietary variables, social support, age, sex) were excluded. The resulting analytical sample consisted of 855 individuals aged 15–25 years.

Missing data were generally low across variables. Age and sex had no missingness, and missingness for physical activity, dietary variables and social support remained below 2% for all measures. Given these minimal levels, a complete-case analysis was conducted and no imputation procedures were applied.

### Variables

The dependent variables were clinically diagnosed anxiety and depression, defined as having received a medical diagnosis of either condition. In the regression analyses, the outcome was coded as the absence of the condition (i.e., being anxiety-free or depression-free).

Personal and behavioural characteristics included categorical factors—sex (men as the reference category), dietary habits (daily consumption of red and processed meats; use of olive oil for cooking), and perceived social support. Social support was measured using the three-item Oslo Social Support Scale (OSSS-3) included in ESCA, which assesses the number of close contacts, perceived interest from others, and availability of practical help. Scores range from 3 to 14 and were categorized as low (3–8), medium (9–11), and high (12–14), with no social support as the reference category. Age and frequency of vigorous physical activity in the previous week were included as continuous variables.

Physical activity was assessed with the question: “During the last 7 days, how many days did you engage in vigorous physical activity?” Vigorous activity was defined as activities requiring substantial physical effort and causing much heavier breathing than normal (e.g., lifting heavy objects, digging, aerobic exercise, fast cycling). Only activities lasting at least 10 consecutive minutes were considered.

Olive oil use was coded as 1 if the participant reported using olive oil as the main fat for cooking and 0 otherwise, following ESCA’s exact method for data collection. Red and processed meat consumption was originally recorded in three categories (none; less than one serving per day; one serving or more per day) and was dichotomized into any daily consumption versus none. One serving corresponded to approximately 100–150 g.

### Statistical analysis

Descriptive analyses summarized sociodemographic and behavioural characteristics. Percentages were calculated using the number of participants with available data for each variable. Chi-square and t-tests were used to explore bivariate relationships.

Logistic regression models were estimated to examine the associations between explanatory variables and anxiety or depression, controlling for age and sex. Four models were specified: two predicting the probability of being anxiety-free (Models A1 and A2) and two predicting the probability of being depression-free (Models D1 and D2). Models A1 and D1 included only main effects, whereas Models A2 and D2 additionally included an interaction term between sex and physical activity. Adjusted odds ratios (AORs) and 95% confidence intervals (CIs) were calculated, with statistical significance set at *p* < 0.05. Model fit was compared using the Akaike Information Criterion (AIC), with lower values indicating better relative fit.

The ESCA dataset is anonymized and publicly available. The study complies with the ethical standards of the Declaration of Helsinki, and no additional ethical approval was required for this secondary data analysis.

All analyses were conducted in RStudio (version 2025.05.1) using the packages rio [4], dplyr [18], haven [19], descr [2], and RcmdrMisc [7].

## Results

A total of 855 participants aged 15–25 years were included in the analysis (mean age 19.6 years, SD 3.1; 51.5% men and 48.5% women). Overall, 85.7% reported anxiety-free and 93.2% depression-free. Cooking with olive oil was reported by 89.7% of participants, and 28.7% reported not consuming red/processed meat daily. Just over half of the participants (52.1%) reported strong perceived social support, 43.2% moderate support, and 4.8% no support. The mean frequency of vigorous physical activity during the previous week was 1.6 days (SD 2.1) (Table 1).

**Table 1:** Descriptive analyses

Characteristic	n	%	Depression-free^1^		Anxiety-free^1^	
			*n* = 797	% = 93.2	*n* = 733	% = 85.7
Sex						
Men	440	51.5	420	95.5	401	91.1
Women	415	48.5	377	90.8^**^	332	80.0^***^
Used olive oil						
No	86	10.3	83	96.5	80	93.0^*^
Yes	752	89.7	799	93.0	640	85.0
Red/processed meat						
No	242	28.7	232	95.9		
Yes	600	71.3	555	92.5^†^		
Social support						
None	39	4.8	34	87.2		
Medium	354	43.2	328	92.7		
Strong	427	52.1	406	95.1^†^		
Age (mean, sd)	19.6 (3.1)		19.5 (3.1)^***^		19.4 (3.1)^***^	
P. Activity (mean, sd)	1.6 (2.1)		1.7 (2.1)^**^		1.7 (2.1)^***^	

^†^*p* < 0.1

^1^Percentages are based on available data for each variable; denominators may vary due to missing values

^*^p < 0.05; ^**^p < 0.01; ^***^p < 0.001

Bivariate analyses showed significant differences in mental health outcomes according to sex, age, and physical activity. Women were less likely to be depression-free and anxiety-free compared to men (*p* < 0.01 and *p* < 0.001, respectively). Younger participants and those reporting lower levels of vigorous physical activity were more likely to report anxiety and depression. Perceived social support showed a marginal association with depression status (*p* < 0.1), with higher proportions of depression-free individuals among those reporting strong support. A modest association was also observed between olive oil use and anxiety status (*p* < 0.05) (Table 1).

In the models without the interaction term (A1 and D1), several consistent patterns emerged (Table 2). Increasing age was associated with a lower probability of being free from anxiety (OR = 0.89, 95% CI: 0.83–0.96) and depression (OR = 0.88, 95% CI: 0.80–0.97), Indicating that younger participants were more likely to report being free of anxiety and depression. Sex was also a significant predictor: compared to men, women were less likely to be anxiety-free (OR = 0.42, 95% CI: 0.27–0.66) and free of depression (OR = 0.50, 95% CI: 0.27–0.94). Higher levels of vigorous physical activity were associated with better mental health outcomes (anxiety: OR = 1.15, 95% CI: 1.02–1.29; depression: OR = 1.20, 95% CI: 1.00–1.44). Similarly, not consuming red or processed meat was positively associated with better mental health (OR = 1.79, 95% CI: 1.07–2.98 for anxiety; OR = 2.36, 95% CI: 1.07–5.21 for depression). Strong social support was significantly associated with being anxiety-free (OR = 3.46, 95% CI: 1.53–7.82) while the association with depression did not reach statistical significance (OR = 2.74, 95% CI: 0.92–8.15). The negative association observed for olive oil use with anxiety (OR = 0.41, 95% CI: 0.17–1.00) likely reflects residual confounding or measurement limitations rather than a substantive effect.

**Table 2:** Logistic regression models

	**Adjusted Odds Ratio (CI)** **Anxiety**		**Adjusted Odds Ratio (CI)** **Depression**	
**Variables**	**Model A1**	**Model A2**	**Model D1**	**Model D2**
Age (years)	0.89 (0.83–0.96)^***^	0.89 (0.83–0.95)^***^	0.88 (0.80–0.97)^***^	0.88 (0.80–0.96)^***^
Female (Ref.cat. Male)	0.42 (0.27–0.66)^***^	0.57 (0.34–0.96)^**^	0.50 (0.27–0.94)^**^	0.79 (0.40–1.57)
Intense physical activity	1.15 (1.02–1.29)^**^	1.32 (1.09–1.59)^***^	1.20 (1.00–1.44)^**^	1.66 (1.13–2.44)^**^
Consume olive oil (Ref.cat. Not consuming)	0.41 (0.17–1.00)^***^	0.41 (0.17–0.99)^**^	0.52 (0.15–1.76)	0.52 (0.15–1.76)
Not eating red meat (Ref.cat. Eating)	1.79 (1.07–2.98)^**^	1.78 (1.06–2.99)^**^	2.36 (1.07–5.21)^**^	2.33 (1.05–5.18)^**^
Medium social support (Ref.cat. None)	1.88 (0.84–4.20)	1.87 (0.84–4.15)	1.76 (0.60–5.16)	1.80 (0.62–5.22)
Strong social support (Ref.cat. None)	3.46 (1.53–7.82)^***^	3.48 (1.55–7.81)^***^	2.74 (0.92–8.15)	2.83 (0.96–8.4)^*^
Female × physical activity		0.78 (0.61–0.99)^**^		0.61 (0.39–0.94)^**^
AIC	618.53	616.30	371.92	367.63

^*^p < 0.10, ^**^p < 0.05, ^***^p < 0.01

When the interaction between sex and vigorous physical activity was included (Models A2 and D2), the anxiety model remained largely consistent. The interaction term indicated that the positive association between physical activity and being anxiety-free was weaker among women compared to men (OR = 0.78, 95% CI: 0.61–0.99). While physical activity remained significantly associated with anxiety-free status, the magnitude of the effect differed by sex. For depression, including the interaction showed a clearer differential pattern. Vigorous physical activity was strongly associated with being free from depression among men (OR = 1.66, 95% CI: 1.13–2.44), while the interaction term suggested a weaker association for women (OR = 0.61, 95% CI: 0.39–0.94). Once the interaction was included, sex was no longer a significant predictor, suggesting that observed gender differences in depression may reflect differential responses to vigorous activity rather than direct effects of sex.

The AIC values slightly decrease when the interaction term is included (A1 (618.5) → A2 (616.3) and D1(371.92) → D2(367.63)), suggesting a modest improvement in model fit, especially for the depression model.

## Discussion

The present study explored the factors associated with anxiety and depression among adolescents and young adults in Catalonia, focusing on lifestyle behaviors and social determinants. Using data from the Catalan Health Survey, we found that younger age, male gender, intense physical activity and lower consumption of red or processed meat were associated with better mental health, while strong perceived social support emerged as a robust protective factor. In this line, the findings reinforce the importance of lifestyle habits in shaping the mental health of young people in Catalonia.

Regular physical activity was associated with lower levels of anxiety and depressive symptoms, which is consistent with evidence highlighting both neurobiological pathways (e.g., regulation of endorphins, cortisol, inflammatory markers) and psychosocial mechanisms such as enhanced self-esteem, social connectedness, and perceived competence [3, 5, 11]. These mechanisms jointly support the well-established protective role of physical activity in adolescent mental health.

Beyond this general pattern, our findings reveal a gender-differentiated association. As widely documented in the literature, girls reported higher levels of anxiety and depressive symptoms [1, 12, 13], likely reflecting a combination of hormonal, psychosocial, and cultural factors. Importantly, the interaction between sex and physical activity observed in our models indicates that the protective effect is stronger in boys. This aligns with research suggesting that males tend to benefit more from vigorous exercise, whereas girls may respond more favorably to moderate-intensity or socially oriented activities [15]. Thus, rather than contradicting the general protective role of physical activity, our results refine it by showing that its magnitude and form may vary across genders. Additionally, the inverse association between age and the probability of being free of anxiety suggests increasing vulnerability as adolescents transition into adulthood, potentially due to intensified academic and work-related demands.

Dietary habits also showed notable associations. Participants who reported not eating red or processed meat had a higher likelihood of being free from anxiety and depression, supporting evidence linking high meat intake with inflammation and adverse mood symptoms [9, 10]. In contrast, olive oil use showed no consistent association with anxiety or depression.. This unexpected finding may reflect residual confounding, measurement variability, or sample imbalance rather than a true negative effect. A consistent association has been documented in the literature between Mediterranean dietary patterns—typically rich in olive oil—and mental health [9].

Beyond behavioral determinants, perceived social support emerged as one of the factors most strongly associated with better mental health. Individuals with strong support networks were more than three times as likely to be free from anxiety compared with those reporting no support. Consistent with social epidemiological theory, strong interpersonal networks may be commonly associated with lower stress and higher resilience during adolescence and early adulthood—a developmental period characterized by psychological vulnerability [14, 16]. Thus, youth programs and community-based interventions that enhance peer and family support could therefore have significant preventive value.

Beyond their relevance at the individual level, these findings have broader implications for public health strategies, particularly within global efforts to improve mental well-being among adolescents and young adults. The identification of modifiable lifestyle and social determinants—such as physical activity, dietary habits, and perceived social support—aligns with the objectives of the United Nations Sustainable Development Goal 3 (Good Health and Well-being), which emphasizes the prevention of non-communicable diseases and the promotion of mental health across the life course. Population-based evidence such as that provided by the ESCA survey can help inform prevention-oriented policies and interventions targeting adolescents and young adults, a group particularly vulnerable to the onset of anxiety and depressive disorders.

This study has several strengths, including the use of a large, population-based dataset and the integration of behavioral and social determinants within a single analytical framework. However, limitations should be acknowledged: the cross-sectional design precludes causal inference, and self-reported measures may be affected by recall or social desirability bias. Moreover, the binary nature of dietary variables may not fully capture the complexity of Mediterranean eating patterns.

Future studies should employ longitudinal designs and detailed assessments of diet and physical activity—potentially incorporating biomarkers—to clarify the causal pathways linking lifestyle factors and mental health.

Taken together, these findings emphasize the multidimensional nature of mental health among young people. Interventions promoting physical activity, healthy diets, and strong social relationships may serve as effective strategies to prevent or reduce anxiety and depressive symptoms. Public health policies should consider gender-specific approaches and the combined influence of behavioural and social determinants when addressing youth mental health in Catalonia.

## Conclusion

This study highlights the critical role of lifestyle and social factors in shaping mental health among young people in Catalonia. Regular intense physical activity, reduced consumption of red and processed meats, and strong perceived social support were identified as factors associated with lower levels of anxiety and depression, with gender differences observed in the strength of these associations.

These findings underscore the importance of integrated public health strategies that promote physical activity, nutritional education, and social inclusion from adolescence onwards. Tailored, gender-sensitive interventions in schools, universities, and community settings could contribute to reducing the burden of mental health problems among youth.

Future research should further explore the causal mechanisms underlying these associations and evaluate intervention programs that combine behavioral and social approaches to mental well-being.

## Data Availability

The data that support the findings of this study are available from Enquesta de Salut de Catalunya, but restrictions apply to the availability of these data, which were used under license for the current study and so are not publicly available. Data are, however, available from the authors upon reasonable request and with permission of Enquesta de Salut de Catalunya.

## Footnotes

1 https://scientiasalut.gencat.cat/handle/11351/1566/browse?resetOffset=true&type=dateissued&order=DESC&rpp=100
